# Notes and News

**Published:** 1896-05-16

**Authors:** 


### NOTES AND NEWS.

(Collected and Communicated.)

### HOSPITAL MEETINGS.

The Annual General Meeting of Governors of the British Home for Incurables, Streatham, will be held at the City Terminus Hotel, Cannon Street, on Thursday, May 21st, at 12 o'clock.

The Annual General meeting of Governors of the Hospital for Women, Soho Square, will be held at the Hospital on Thursday, May 21st, at 3 p.m.

The Annual Meeting of the Governors of the Victoria Hospital for Children will be held on Wednesday, May 20th, at 5 o'clock, in the Board Room of the Hospital.

The adjourned Annual Court of the Governors of the Royal Orthopædic Hospital will be held at the Hospital on Thursday, May 21st, at 4 p.m.

The new wing of the Derbyshire Royal Infirmary, erected at the cost of Mr. Walter Evans, will shortly be completed and ready for occupation.

We are glad to hear that Aberdeen is likely shortly to fall into line with other Scotch towns, and to adopt a system of working-men's subscriptions to its hospitals.

Her Majesty the Queen has graciously consented to become an annual subscriber of ten guineas to Queen Charlotte's Lying-in Hospital. H.R.H. the Duke of Cambridge has made a further donation of £5.

The Lord Chief Justice of England (Lord Russell of Killowen) will preside at the festival dinner of the London Society for Teaching the Blind, to be held at the King's Hall, Holborn Restaurant, on the 18th inst.

Mr. Passmore Edwards, on the 28th ult., opened the Cottage Hospital (eight beds) which he has presented to the town of Liskeard. The hospital is similar in design to the other cottage hospitals erected through Mr. Passmore Edwards' munificence.

The co-operative system has proved very disadvantageous to the retail druggists. In Chicago they are taking prompt action for their own protection. Four hundred druggists have combined together, to form a company; they have leased a manufactory, and all the stores in the combination will procure their supplies from the factory at such low prices that they hope to undersell all the drug departments in the co-operative stores.

The formal opening of the new wing (which, it is estimated, cost £3,100) of the West Kirby Convalescent Home for Children took place on the 30th ult.

Sir Benjamin Baker, K.C.M.G., LL.D., F.R.S., President of the Institution of Civil Engineers, will preside at the annual dinner of old students of King's College, London, to be held at the Holborn Restaurant on June 29th.

Sir William Broadbent, the senior physician, has consented to preside at a festival dinner to be held on June 17th at the Whitehall Rooms, Hotel Metropole, with the object of raising the £12,400 necessary to complete the new out-patients' department of St. Mary's Hospital.

Mr. J. J. Colman having some months back offered to present the Jenny Lind Infirmary, Norwich, with the site for its new building, as a memorial to the late Mrs. Colman, this offer has been accepted. The committee of management has been empowered to prepare plans for the hospital and to take the necessary steps for collecting funds.

The first annual report of the Clinical Research Association shows how necessary such an institution is under modern conditions of medical practice, and how acceptable to the profession has been the aid it offers, for it appears that during the first twelve months that the laboratory was opened the number of investigations conducted therein amounted to 5,227. It might have been imagined that, with the education which the present generation of medical men have received, they would have been quite able to conduct such investigations for themselves; but, as a fact, scientific methods advance so rapidly, and the complexity of the instruments employed in scientific research increases at such a rate, that it is next to impossible for any man engaged in active practice to obtain the full advantage of the most recent science in the diagnosis of his cases without the assistance of such an association as this.

Besides various articles bearing on the more specialised departments of science, the month's *Science Progress* contains an interesting address by Professor Frankland, F.R.S., on "The Past, Present, and Future Water Supply of London." He points out the efficacy of complete filtration, and maintains that as a factor in the production of a pure water supply perfect filtration is of greater importance than the purity of the source from which the water is drawn. "The line of demarcation between the past and the present water supply of the metropolis is . . . . to be drawn, not when the intakes of the river companies were removed to positions beyond the possibility of pollution by the drainage of London, but must be drawn at the time when efficient filtration was finally secured and ever since maintained, that is to say in the year 1884." His conclusion is that "for half a century at least we have at our doors, so to speak, an ample supply of water, which for palatability, wholesomeness, and general excellence will not be surpassed by any supply in the world." The interest of this article is considerably increased by the fact that just at the present moment the innocence and harmlessness of London water is being seriously called in question by various metropolitan sanitary authorities.

